# Overcoming Barriers: A Comprehensive Review of Chronic Pain Management and Accessibility Challenges in Rural America

**DOI:** 10.3390/healthcare12171765

**Published:** 2024-09-04

**Authors:** Maxwell B. Baker, Eileen C. Liu, Micaiah A. Bully, Adam Hsieh, Ala Nozari, Marissa Tuler, Dhanesh D. Binda

**Affiliations:** 1 Department of Anesthesiology, Boston University Chobanian & Avedisian School of Medicine, Boston, MA 02118, USA; ecliu25@bu.edu (E.C.L.); mbully@bu.edu (M.A.B.); ala.nozari@bmc.org (A.N.); marissa.tuler@bmc.org (M.T.); ddb96@bu.edu (D.D.B.); 2 Larner College of Medicine, University of Vermont, Burlington, VT 05405, USA; 3 Department of Anesthesiology and Pain Medicine, University of Toronto, Toronto, ON M5S 1A1, Canada; adam.hsieh@mail.utoronto.ca; 4 Department of Anesthesiology, Montefiore Medical Center, Bronx, NY 10467, USA

**Keywords:** chronic pain, rural healthcare, pain management, healthcare disparities, healthcare access, integrated care models

## Abstract

In the United States (U.S.), chronic pain poses substantial challenges in rural areas where access to effective pain management can be limited. Our literature review examines chronic pain management in rural U.S. settings, identifying key issues and disparities. A comprehensive search of PubMed, Web of Science, and Google Scholar identified high-quality studies published between 2000 and 2024 on chronic pain management in the rural U.S. Data were categorized into thematic areas, including epidemiology, management challenges, current strategies, research gaps, and future directions. Key findings reveal that rural populations have a significantly higher prevalence of chronic pain and are more likely to experience severe pain. Economic and systemic barriers include a shortage of pain specialists, limited access to nonpharmacologic treatments, and inadequate insurance coverage. Rural patients are also less likely to engage in beneficial modalities like physical therapy and psychological support due to geographic isolation. Additionally, rural healthcare providers more often fulfill multiple medical roles, leading to burnout and decreased quality of care. Innovative approaches such as telehealth and integrated care models show the potential to improve access and outcomes. Our review highlights the need for increased telehealth utilization, enhanced provider education, and targeted interventions to address the specific pain needs of rural populations.

## 1. Introduction 

Chronic pain is a pervasive and complex issue that impacts many patients in the United States (U.S.). Defined by the International Association for the Study of Pain (IASP) as pain lasting three or more months, chronic pain was experienced by an estimated 20.9% of all U.S. adults in 2021 [1]. Chronic pain has a detrimental impact on patients’ quality of life and has been linked to depression, dementia, higher suicide risk, and substance use [2,3,4,5]. Of those with chronic pain, many suffer from constant pain, affecting one’s ability to conduct activities of daily living (ADLs), as well as the ability to work and earn income. The severity of pain that individuals experience is diverse, with one study finding about half of individuals experience mild pain (1–5 on a 1–10 pain scale, 0 meaning no pain and 10 meaning worst pain they have ever felt), one-fifth experiencing moderate pain (6–7 on pain scale), and one-third experiencing severe pain (8–10 on pain scale) [6]. Arthritis is a leading cause of chronic pain, which accounts for approximately one-third of patients [6]. Other prevalent causes include sciatica and vertebral pain (17.6%), traumatic injury (11.6%), and muscle pain (6.3%) [6]. Across subpopulations in the U.S., levels of intensity and frequency of pain are similar. However, disparities in prevalence become evident across geographic regions, socioeconomic status, sexual orientation, and chronic comorbidities [7]. For example, data from the Behavioral Risk Factor Surveillance System in 2018 shows that the prevalence of chronic pain in rural populations is 30.9%, significantly higher than the prevalence of 19.6% observed in urban populations [1]. Additionally, individuals with chronic pain in rural areas are 3.5 times more likely to have neuropathic pain than those in urban areas [6]. This disparity can partially be attributed to the increased age of the rural population, which on average is 43 years old, compared to 36 years old in urban areas [8]. Older populations in rural areas have been observed to work at older ages than those in urban areas [9]. Furthermore, rural residents are more likely to have lower annual salaries and occupations that demand greater physical exertion than their urban counterparts [8].

When considering healthcare costs, work missed, and decreased wages, it is estimated that chronic pain expenditures in the U.S. range from $560 to $635 billion U.S. dollars per year [10]. Chronic pain not only impacts the productivity and economic strength of the U.S. but also has negative effects on the health and well-being of rural populations. Patients in rural settings are more likely to experience limitations due to chronic pain compared to urban patients [11]. Additionally, patients in rural areas can be more susceptible to healthcare shortages and poverty, tend to be located further from healthcare services, have a higher incidence of comorbidities, and are more likely to engage in unhealthy behaviors such as smoking [6,7]. Rural populations are also more likely to be older than urban populations, with an increasing percentage of people aged 65 years and older [12]. Thus, the consequences of chronic pain grow increasingly dire with an aging population and necessitate improved strategies for management [1,9].

Chronic pain is ideally managed using a multimodal, interdisciplinary therapeutic approach. These therapies are typically overseen by interdisciplinary teams at larger urban institutions that include specialists in pain medicine, physical therapy, psychiatry, pharmacy, and primary care. Care is coordinated among all these members, and progress updates ensure that the patient’s total well-being is considered [13]. In rural areas, care is often distributed quite differently as chronic pain is largely managed by singular primary care providers (PCPs) [14]. A minority of patients in rural areas receive care from or have access to specialty pain clinics, which places a high burden on PCPs [15]. This care disparity has led to rural residents being less likely to receive nonopioid and nonmedical therapies [16]. As a result, less effective pain management leads to increased substance use of opioids, as well as cannabis, alcohol, and nicotine [5]. Certain comorbidities such as decreased renal or hepatic function, stomach ulcerations, cardiac diseases, and cognitive disorders further limit available medical interventions to providers and necessitate careful medication selection and dose titration [17].

The primary goal of this literature review was to assess the current understanding of chronic pain management in rural areas of the U.S., including the nature of chronic pain, its management strategies, and the associated healthcare disparities and access issues.

## 2. Materials and Methods

We performed a comprehensive search of PubMed, Web of Science, and Google Scholar on 15 January 2024. We employed a set of carefully chosen keywords and phrases to capture the most relevant studies. These terms included “chronic pain”, “rural healthcare”, “pain management”, “healthcare disparities”, “healthcare access”, and “United States”. Several keywords were selected to encompass a broad range of research focusing on the management of chronic pain in rural settings within the U.S. Published articles between 2000 and 2024 were reviewed in order to focus on recent research and ensure up-to-date insights. Articles were included if they specifically addressed chronic pain management within rural areas of the U.S., focusing on clinical practices, access to care, patient outcomes, or health policy. Eligible studies must have been conducted in populations residing in rural regions, as defined by the U.S. Census Bureau or other recognized rural classification systems, and must have provided clear geographical details confirming the rural context. Articles were excluded if they primarily discussed urban or suburban populations, lacked a clear focus on chronic pain management strategies or outcomes in rural settings, did not directly relate to the topic, or were not originally published in English.

The evaluation process for selecting studies was rigorous and included an initial screening of titles and abstracts by MBB, ECL, and MAB for relevance to the review’s objectives. Articles that met the preliminary criteria were then thoroughly examined. Each author decided which articles to include at their discretion. A focus was placed on studies that provided robust evidence, such as randomized controlled trials, prospective cohort studies, systematic reviews, and meta-analyses. Each selected article was meticulously analyzed for crucial information, including the authors, publication year, study design, sample size, and key findings. This information was categorized into four thematic areas: Challenges in Managing Chronic Pain in Rural Settings, Current Strategies for Pain Management, Gaps in Research and Practice, and Innovative Approaches and Future Directions. We synthesized a logical and coherent representation of the literature on chronic pain management in rural U.S. settings. We then examined the consistency of our findings, explored discrepancies, and drew conclusions based on the overall evidence. 

## 3. Results

Our search yielded 14 studies from 2004 to 2024 that met the inclusion criteria (Table 1). Rural locations in the U.S. included Alaska, Alabama, Idaho, Iowa, Michigan, Montana, New York, North Carolina, Washington, Wisconsin, and Wyoming. Key quotes capturing the main focus and core themes of each article were selected and included to summarize their essential arguments (Table 1). An analysis of these studies revealed several challenges across different themes, including clinician burnout, decreased engagement with pain modalities, multipharmacy, distrust in healthcare, nonevidence-based pain management plans, pain management by nonspecialists, socioeconomic factors, social and cultural values, and increased opioid prescribing patterns. Notably, there was a higher prevalence and severity of chronic pain in rural populations [8,14].

### 3.1. Economics of Care

While just 8% of the U.S. population live in areas served by pain specialists, 21% of the U.S. population live in rural areas, which also report lower median full-time salaries (6% to 12% lower) and higher uninsured rates (12.3% vs. 10.1%) when compared to urban residents [8,9]. Given that rural residents report working later into their lives in jobs with physically demanding roles such as in agriculture and/or working with heavy machinery, the risk of both chronic conditions from overuse and a traumatic injury, both leading causes of chronic pain, is greatly increased [14].

### 3.2. The Role of the Clinician and Healthcare Systems

**Figure 1:**
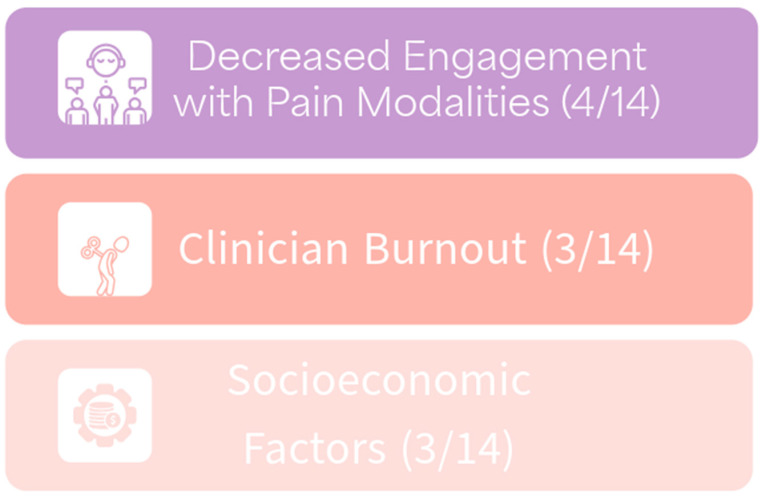
The three most common themes characterizing literature about chronic pain management in rural America.

The shortage of healthcare providers in rural medicine remains a widespread issue in the U.S. and also worldwide. Healthcare providers working in rural settings often fulfill multiple roles, primarily working in solo practice [18,19]. They report feeling burnt out, leading to a decreased ability to make system-based improvements to healthcare delivery [18,19]. Common themes of burnout, decreased engagement, and socioeconomic factors were covered by three or more studies (Figure 1). 

The pressures of attempting to fill the needs of an underserved community can be overwhelming for providers and affect the quality of care. A 2008 study found many residents in rural Iowa nursing homes were treated with medications that were nonadherent with 1998 American Geriatrics Society (AGS) evidence-based guidelines [20]. For example, propoxyphene, which was not an AGS-recommended opioid, was prescribed to 10.7% of residents, while 33% of residents experiencing daily pain received nonscheduled or as-needed medications [20]. Furthermore, nine of the residents surveyed reported that the pain medications they received negatively clouded their cognition [20]. Not only did these nursing home residents receive inadequate pain regimens, but they more frequently experienced complications from the medications prescribed. Rural populations could therefore experience increased complications and decreased efficacy of pain procedures.

Disparities also exist in specialist accessibility. Critical access hospitals, which are defined as rural hospitals with ≤25 inpatient beds and located >35 miles from other hospitals, comprise 61% of hospitals in the rural U.S. [21]. While 15% of these hospitals advertised chronic pain management services, pain medicine physicians only practiced at 5% of them [21]. Additionally, pain physicians were found to perform only around 26% of chronic pain procedures in critical access hospitals, with the majority of pain procedures being performed by nonspecialists [21]. Engagement with different pain modalities was lower among rural residents, who were less likely to use nonmedication treatments [11,22].

### 3.3. The Impact of Race, Ethnicity, and Cultural Values

Socioeconomic factors such as race and ethnicity, education, and cultural values also shape care delivery in rural environments. A study on pain management in Alabama found that rural populations are more likely to be unemployed and have higher poverty and lower literacy rates compared to urban residents [23]. Black/African American patients with low literacy reported higher pain intensity and greater interference with daily tasks [23]. Other research has found Black and Hispanic patients continue to be prescribed fewer opioids and analgesics regardless of their pain [23]. Rural patients were also found to treat themselves first and were more likely to hold stigmatized views of organized care, instead placing a higher value on personal autonomy [24]. Rural Black/African Americans had decreased access to pain clinics [25], and cultural values often favored self-reliance over medical intervention [24]. Distrust in healthcare arose from a lack of transparency in healthcare decisions and poor communication about self-treatment choices [26,27].

### 3.4. Opioids

The careful use of opioids in chronic pain care is essential to provide adequate analgesia while balancing the risks of opioid use disorder (OUD). One study noted that nonrecommended opioids were often prescribed in rural elderly populations, and evidence-based guidelines were inconsistently implemented, resulting in inappropriate and suboptimal pain management [20]. Other research has found rural residency alone as a strong predictor of higher opioid prescription rates, with individuals diagnosed with depression being more likely to receive opioids for chronic pain [28,29]. Social loneliness was also more prevalent among those in chronic pain self-management programs [30]. The cultural belief in self-reliance and reluctance to seek medical help unless severely impaired influenced rural residents’ engagement with pain management [24,27].

**Table 1:** Challenges regarding the delivery of chronic pain management in rural areas.

Author	Study Type	Population	Outcomes	Location	Theme	Selected Quotes	Page Number
Brunner 2022 [30]	Cross-sectional observational	Rural residents who enrolled in available workshops	Experiencing chronic pain is associated with increased social loneliness scores.	New York	Socioeconomic factors	“Those enrolled in the chronic pain self-management program reported higher levels of social loneliness than those enrolled in the other programs.”	1299
Day 2020 [23]	Cross-sectional qualitative	Rural residents with chronic pain from three Alabama counties	Race is associated with pain intensity and pain interference, with African Americans experiencing higher scores of each when compared with Whites.	Alabama	Socioeconomic factors	“Results indicated that race uniquely predicted pain outcomes such that African-Americans reported significantly higher pain intensity and pain interference ratings in comparison to White Americans… Within this context, it is of particular interest that race was also associated with primary literacy; African-Americans obtained significantly lower reading scores than White Americans.”	467
Decker 2009 [20]	Retrospective observational	Residents of rural nursing homes in Iowa	There is poor adherence to evidence-based guidelines in managing chronic pain in rural nursing homes.	Iowa	Nonevidence-based pain management plans	“Propoxyphene, not an AGS-recommended opioid, was also prescribed for 23 (10.7%) residents. Of the 70 (32.6%) residents expressing daily pain, 23 (32.9%) received no scheduled or pro re nata (PRN) analgesics… The findings suggest that the 1998 AGS evidence-based guideline for the management of chronic pain is inconsistently implemented.”	58
Elhakim 2019 [21]	Cross-sectional observational	Critical access hospitals	Only a fraction of critical access hospitals offer interventional pain procedures by pain medicine specialists, indicating a gap in access to specialized care.	Iowa	Pain management by nonspecialist	“Pain medicine physicians were listed as providing care at a very small percentage (≅5%) of the critical access hospitals. However, many more critical access hospitals (≅15%) publicly included interventional procedures to treat chronic pain as a service. Pain physicians were the minority of the clinicians performing the procedures (≅26%).”	53
Gessert 2015 [24]	Systematic review	Rural populations from the United States, Canada, and Australia	Rural populations often define health in terms of functional independence, emphasizing the ability to work and be self-reliant	United States, Canada, Australia	Social and cultural values Decreased engagement with pain modalities	“Rural residents expressed the belief that a “work hard, eat hard” attitude kept them healthy despite the stress of their work and living in a rural environment.” “Additionally, rural residents would only seek a physician’s help if physical functioning was severely impaired.”	380
Kapoor 2014 [29]	Retrospective observational	Rural residents, primarily female and African American	Depressive symptoms significantly influenced healthcare utilization among rural residents with chronic pain.	Alabama	Increased opioid prescribing patterns	“It is noteworthy that those with a clinical diagnosis of depression were more than three times likely to receive opioid prescriptions for their chronic pain.”	2887
Mares 2023 [25]	Cross-sectional observational	U.S. military veterans with chronic pain who presented to the VA in 2018	Decreased pain clinic visits were associated with an increased use of the emergency department and urgent care.	United States	Socioeconomic factors Decreased engagement with pain modalities	“Black Americans were less likely to receive pain clinic visits (aRR = 0.87, CI: 0.86–0.88).” “Rurality further decreased the likelihood of Black Americans visiting a pain clinic.”	595
Parchman 2020 [18]	Qualitative interview-based	Staff and clinicians from 6 rural primary care organizations across Washington, Wyoming, Alaska, Montana, and Idaho	Facilitators and barriers to system-wide changes in opioid prescribing were identified.	Washington, Wyoming, Alaska, Montana, Idaho	Clinician burnout	“In these rural settings, clinicians and staff often worked in multiple roles and covered for unfilled positions.”	428
Parlier 2018 [19]	Narrative review	Medical students, resident physicians, and rural attending physicians	Many different factors influence the recruitment and retention of physicians in rural areas.	United States, Canada, Australia	Clinician burnout	“The main stressors for rural physicians include low reimbursement, insufficient practice management skills, work-life imbalance, heavy workload, too frequent calls, isolation, and inadequate professional support.”	135
Prunsuke 2014 [28]	Cross-sectional observational	9,325,603 U.S. adults seen in primary care clinics in 2010	Rural and non-Caucasian residents had significantly higher odds of being prescribed opioids for NMCP.	United States	Increased opioid prescribing patterns	“First, rural residents had higher odds of having an opioid prescription than similar non-rural adults. Rural residency was the strongest predictor for having an opioid prescription and a diagnosis for NMCP.”	567
Qudah 2022 [26]	Participatory design approach	Patients managing chronic pain + healthcare providers in rural Southeastern Wisconsin	Key challenges related to opioid use and chronic pain management in a rural community were identified.	Wisconsin	Clinician burnout Distrust in healthcare	“Providers are under significant pressure to achieve high patient satisfaction ratings, limit the loss of patients, and refer patients with OUD to treatment despite institutional policies that facilitate such referral. Each of these factors shape the treatment decisions made by providers. “Providers are then viewed as unprofessional and unempathetic by patients who likely are not aware of the myriad of forces that are influencing provider decision-making “behind the scenes”.”	106
Rafferty 2021 [6]	Participatory survey design	North Carolina participants of the 2018 Behavioral Risk Factor Surveillance System	Rural and suburban residents have a higher prevalence of chronic pain compared to urban areas and are less likely to use nonmedication therapies.	North Carolina	Decreased engagement with pain modalities Multipharmacy	“Adults with chronic pain in suburban and rural areas were less likely to use nonmedication treatments” “... and less likely to use 3 or more types of treatments compared with adults in urban areas.”	N/A
Rodgers-Melnick 2024 [22]	Cross-sectional observational	7114 adults with chronic pain from the 2019 National Health Survey	The study identified several factors associated with IHM and nonpharmacologic chronic pain management.	United States	Decreased engagement with pain modalities	“Chronic pain is more prevalent in rural areas, yet we found that non-metropolitan residence was associated with reduced odds of engagement in nonpharmacologic and IHM modalities.”	261
Vallerand 2004 [27]	Cross-sectional observational	Rural patients from Michigan with the majority being women	A significant portion of the rural population relies on self-treatment for pain management.	Michigan	Multipharmacy Distrust in healthcare Social and cultural values	“Herbal products and supplements, opioid analgesics, and adjuvant analgesics were used by 18–20% of the participants.” “Of concern are the findings that participants reported that only about half of their pain was relieved by their self-treatment choices and that 20% had not informed their primary care practitioners of their self-treatment choices.” “…the rural work ethic and sense of self-reliance often found in rural communities may influence the value placed on education for self-treatment.”	171

Note: IHM, integrative health medicine; OUD, opioid use disorder; AGS, American Geriatric Society; NMCP, nonmalignant chronic pain.

## 4. Discussion

Our literature review highlights numerous factors that contribute to the observed disparity in chronic pain prevalence between rural and urban populations. Physically demanding occupations such as farming, animal husbandry, and heavy machinery operations increase the risk of potential osteoarthritis, traumatic injury, and muscle pain, which are leading triggers for chronic pain. When combined with longer career lengths, the risk of chronic pain is further increased in rural residents [14]. Additionally, rural residents may be more likely to forgo early treatment and medical intervention due to costs associated with care and limited access to health resources, as rural residents report lower incomes than urban residents, which poses a significant hindrance to the seeking of care, not only for pain management but also for preventive care [8]. Such preventive measures may reduce the risk of developing diabetes, alcohol use disorder, and vascular disease, all of which are known etiologies of neuropathic pain [31]. In addition to reduced income, rural residents are also more likely to be uninsured when compared to those living in nonrural counties [31]. Examining all of these factors reveals how important it is to understand and address each one to help address the disparities observed between the prevalence and treatment of chronic pain among geographic regions in the U.S. A continued emphasis on innovation in these care delivery systems could help reduce disparities among rural communities seeking chronic pain services.

### 4.1. Challenges in Managing Chronic Pain in Rural Settings

Challenges from socioeconomic barriers to clinician burnout all affect care delivery and can manifest as disparities between rural and urban populations. Rural populations earn less income, are less likely to be insured, and are more likely to work physically demanding jobs compared to their urban counterparts [8,9,14]. Reduced income and insurance coverage can both prevent a patient from seeking care due to inability to pay, which compounds with the increased chance of chronic pain from a traumatic or chronic injury associated with the enhanced physical demands of the rural workforce. When considered within the context of the shortage of clinicians in rural America, the gap in care delivery becomes apparent.

One contributing factor to the shortage of rural providers is the high prevalence of rural clinician burnout, which stems from filling multiple roles and vacant positions [18], exacerbated by low reimbursement, heavy workloads, and insufficient support [19]. Burnout leads to a decreased ability to make system-based improvements to healthcare delivery [18,19]. The additional pressure to achieve high patient satisfaction and refer patients with OUD further contributed to burnout and perceptions of unprofessionalism [26]. Reduced provider engagement can also affect the quality of care, which has been investigated in rural chronic pain populations in a variety of studies. Nonrecommended opioid administration and suboptimal pain management were common themes observed in one 2008 study investigating rural Iowa nursing home chronic pain care. Residents of the nursing home reported inadequacies in their analgesia, regular medication complications, and effects on their cognition [20].

The challenges of receiving adequate chronic pain management in rural communities are amplified by several demographic factors. Harmful stereotypes, such as the misconception that Black/African American patients have a higher pain tolerance, perpetuated racial biases in healthcare settings, resulting in inequitable and substandard care. Black/African American patients are also less likely to receive care at a pain clinic, a disparity that is even greater for those who live in rural areas [25]. Furthermore, rural populations are more likely to be older, experience loneliness, and have chronic diseases, which are all associated with higher rates of chronic pain and mortality [30,32]. 

Rural populations may have different beliefs regarding health and wellness, requiring tailored management plans. Such patients may be more likely to accept ill health and death as natural phenomena and are less likely to seek help if physical functioning is impaired [24]. Patients who live in nonmetropolitan areas are less likely to engage in nonpharmacologic or psychosocial modalities of pain management that could be potentially beneficial [6,22]. These modalities include yoga, Tai Chi, acupuncture, chiropractic manipulation, massage, and meditation. This lack of engagement may exist due to increased geographic isolation, lack of access to transportation, and limited services in rural areas [22]. Additionally, patients report frustration with insurance reimbursement for these modalities, such as physical therapy due to the limited number of sessions and high copays [26]. All of these factors contribute to distancing rural patients from healthcare systems and instead driving patients to self-treat their pain, potentially using over-the-counter medications and herbal supplements. These beliefs and practices can hinder medication adherence, discourage patients from seeking professional management for chronic pain, and lead to self-medication with alternatives that may increase the risk of adverse drug reactions.

Pain management in rural communities is further complicated by opioid misuse. Rural patients with nonmalignant chronic pain are more likely to have an opioid prescription compared to urban residents [28]. Rural residents with depression and comorbidities are also more likely to receive opioids for chronic pain, independent of pain severity [29]. Among patients with no substance use history, those who are clinically depressed and experiencing comorbid conditions tend to misuse prescription opioids [29]. Other factors that increase the risk of OUD in rural populations include easy access to opioid prescription, lower employment, economic insecurity, and health-related stigma. Inadequate provider knowledge, lack of accessible recovery centers, and reduced availability of medications to treat OUD in rural areas can further exacerbate the effects of OUD [26]. The absence of adequate Food and Drug Administration (FDA) oversight also contributes to inappropriate opioid prescribing patterns, although this has been improving with recent federal regulations on opioid prescribing [26]. However, inadequate communication with pharmacists and restrictive pharmacy chain policies can result in insufficient opioid medication supplies, driving patients to seek these pain medications from alternative sources.

### 4.2. Current Strategies for Pain Management in Rural Areas

**Figure 2:**
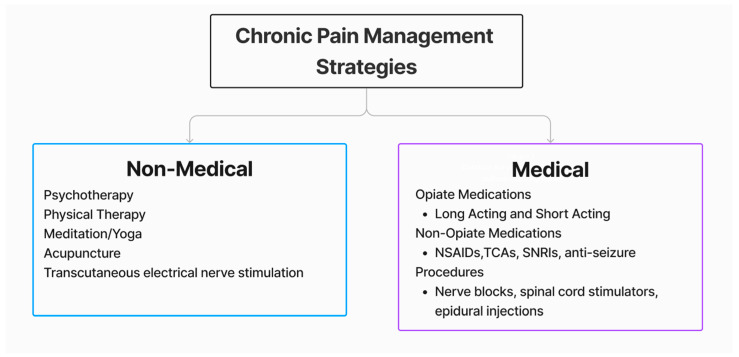
Summary of current evidence-based strategies for chronic pain management.

While opioids are still widely used in chronic pain management, nonpharmacological/interventional therapies, such as therapeutic rehabilitation and exercise, stretching, acupuncture, hydrotherapy, cognitive-behavioral therapy (CBT), mind–body therapy, and transcutaneous electrical nerve stimulation (TENS), are being increasingly used in patient care (Figure 2) [16,17]. Psychotherapy and CBT can help individuals develop personalized techniques to better accept their pain and confront the emotions associated with it [17]. Physical therapy, meditation, and yoga similarly help the patient manage their pain and associated feelings while promoting mobility and activeness. The use of yoga has been associated with less frequent and less intense pain episodes while increasing awareness of body positioning and signals that exacerbate pain [33]. In part, nonmedical approaches allow for changes in cognitions and behaviors toward chronic pain that can provide additional relief from symptoms for patients [33].

Interventional/procedural pain management techniques can also be highly effective but are often reserved for patients who have failed conservative therapies and medications. Many interventional therapies are meant to deliver local anesthetic and/or anti-inflammatory agents at the root source of pain, with common approaches being intra-articular, intramuscular, and intrabursal injections of steroids, which relieve pain at highly targeted locations [17]. Other approaches such as epidural steroid injections have shown some efficacy in reducing radicular pain, while nerve blocks at facet and sacroiliac (SI) joints have been shown to provide short-term management of axial pain [34,35]. Sympathetic blocks provide analgesia when pain is exacerbated by sympathetic responses to stimuli and are commonly administered in the setting of ischemia [36]. Though these various blocks are highly effective, their benefits tend to only last months to a few years and require repeated blocks to maintain analgesia. Newer techniques, such as radiofrequency ablation, spinal cord stimulators, and intrathecal opiate infusion pumps, seek to prolong the duration of the therapeutic window [36].

While multimodal approaches to pain management are more efficacious, many patients in rural areas struggle with access to these services [13,16]. Rural residents are less likely to utilize nonmedical pain therapies, and even when they have access, utilization may be limited, further contributing to enhanced opioid prescriptions in this population [16,37]. Improved access using telecommunication technologies such as telehealth could potentially increase the utilization of nonmedical pain services. However, a recent study by Chen et al. of veterans with chronic pain in rural and nonrural areas found that the use of telehealth did not increase the size of the population utilizing pain services in rural settings [37]. Instead, those already receiving pain management and facing transportation challenges chose to shift to telehealth as an alternative to in-person visits. The reasons for this shift may include hesitancy around interactions with providers in rural communities and challenges associated with transportation and travel to the appointment [38].

Telehealth and virtual care have the exciting potential to address care delivery access in rural and remote areas. The success of telehealth depends on the receptiveness of both patients and providers to this modality. To facilitate its integration, patient-facing pain management education programs focused primarily on rural areas are essential for building patient education and trust. Patient education can be a challenging task. For example, a cross-sectional study found that while the majority of participants who had received pain education were able to adjust their pain management techniques, there was still a significant proportion who did not change their pain cognition or coping strategies [39]. However, those who shifted their management strategies were more likely to experience lower perceived pain. Educating patients about pain management must be designed effectively in order to successfully allow patients to adjust their current strategies and expectations. A combination of robust patient education and thoughtful integration of telehealth services could yield promising benefits in reducing the burden rural PCPs face in managing chronic pain services [6].

### 4.3. Gaps in Research and Practice

There exist numerous opportunities for further research in chronic pain management within rural populations. The majority of our included studies focused on White and Black racial groups. However, according to the Centers for Disease Control and Prevention (CDC), about 40% of Indigenous Americans live in rural areas, with a majority experiencing chronic diseases such as hypertension and diabetes [40]. This disparity necessitates further research on the effects of rurality and other demographic factors on the accessibility and delivery of chronic pain management in additional patient groups, such as Indigenous populations. 

With advancements in remote telecommunication, increased opportunities for working from home, and the recent COVID-19 pandemic requiring sheltering in place, U.S. adults have experienced an increased prevalence of loneliness [30,41]. Factors such as geographic barriers leading to social isolation, poorer health, and socioeconomic disparities may contribute to loneliness in rural populations. Since patients experiencing chronic pain are more likely to report loneliness and isolation, more research should be conducted regarding strategies to mitigate loneliness and identify risk factors in rural populations.

There are also many areas for improvement in clinical practice. A lack of board-certified pain providers, mismarketing of pain services by hospitals, and nonadherence to evidence-based pain management continue to disproportionately impact rural communities [20,21,27]. With a paucity of alternative pain management modalities and limitations set by insurance companies, rural patients are being prescribed opioids at a higher rate than their urban counterparts. This concerning practice, especially given the lack of opioid use support, causes rural communities to suffer not only from inadequate pain management but also from complications surrounding treatment [26]. More research and development is urgently needed regarding safeguards to prevent the aberrant usage of opioid medications, along with the efficacy of current federal regulations regarding prescription patterns.

Complementary pain treatment modalities, such as meditation, Tai Chi, and yoga, are also being utilized less frequently in rural communities, despite playing a role in decreasing chronic pain symptoms and depression [33,34,42,43,44,45,46]. Nonmedical interventions for chronic pain should be offered to rural patients along with education regarding nonpharmaceutical pain modalities. However, with a lack of proper providers, rural patients are likely to have difficulty accessing these therapies [47]. Challenges to retaining complementary pain providers include a lack of available housing, increased commutes, and lack of familiarity with cultural norms [47]. Strategies such as purposeful recruitment of physical therapy students from rural areas, incorporating rural health into the curriculum to improve cultural understanding, and improving telehealth competency to allow greater rural population outreach may help mitigate this staffing gap.

The use of telehealth has steadily grown in an effort to increase healthcare accessibility for patients. Telehealth options, such as virtual physical therapy, have been shown to confer effective and satisfactory results while helping patients reduce the amount of time, money, and distance traveled to see their providers in person [48]. Telehealth utilization has increased faster in rural populations compared to urban populations. However, rural areas have not yet reached the same overall level of utilization [37]. More outreach and education should be conducted to improve rural patient access to telehealth [32]. Virtual options may help to slowly cover some of the gaps of care rural patients face and expand their options for nonopioid interventions. 

Barriers, including the lack of physical exams and the inability to provide procedures virtually, will unfortunately limit the efficacy of telehealth services. With telehealth expansion, patients will need high-speed internet access, digital literacy, and device access, all of which may present challenges for an aging rural population [49]. Rural populations also face limited availability of different cellular, wireless internet, and cable options and may need to spend more money to gain access to telehealth services than their urban counterparts [49]. More assistance programs should be created to help patients access telehealth modalities to decrease the digital health gap. 

Additionally, reimbursement regarding telehealth visits varies widely in the U.S. with coverage protections created during the COVID-19 emergency set to expire [50]. This loss of reimbursement protection may lead to decreased compensation for telehealth visits, de-incentivizing providers from offering virtual options. Insurance coverage of telehealth may also become more variable, leading to financial burdens and confusion for patients. Reimbursement plans should be stabilized as well to help adequately compensate rural providers. Provider training with an emphasis on rural care has not been found to produce increased intention for rural practice [19]. Instead, financial incentives, rural integration, and positive rural exposure should be offered to trainees to help with the retention and promotion of rural practice [19]. Furthermore, specialized pain training for rural providers may help mitigate the pain specialist shortage.

### 4.4. Innovative Approaches and Future Directions

Neuromodulation has significantly advanced over the past two decades, utilizing electrical currents of various frequencies to alter neurotransmitter release and reduce the need for opioids in managing spinal and localized pain [51,52]. Spinal cord stimulation (SCS) has shown high efficacy for spinal, axial, and postoperative pain [51,52]. Targeted neuromodulation techniques include dorsal root ganglion stimulation, peripheral nerve stimulation, and SI joint fusion. Dorsal root ganglion stimulation, approved in the U.S. in 2016, provides relief for up to four specific regions and is effective postoperatively for procedures like mastectomy [53]. Sacroiliac joint fusion treats SI dysfunction by immobilizing the joint with screws and allografts combined with electrical neuromodulation, proving highly effective at reducing pain [51,52]. Innovative pain management techniques for spinal stenosis include percutaneous interosseous spacers and percutaneous lumbar decompression. Percutaneous interosseous spacers reduce complications, procedural time, and postoperative rehabilitation while lowering costs compared to invasive alternatives [54]. The MiDAS trial showed that percutaneous lumbar decompression improves patients’ disability index (ODI) and pain/neuro-ischemic domains (ZCQ domains) [54]. These innovations aim to lower healthcare costs while enhancing patient outcomes.

There are numerous promising innovative therapies in the early stages of development that require further investigation. For instance, low-intensity light-emitting diode (LED) and laser photobiomodulation therapy have shown evidence of musculoskeletal pain reduction by reducing action potentials from pain neurons [55]. There has been an expansion of new ultrasound-guided techniques that have also shown efficacy in pain management [53]. Additionally, cholecystokinin-2 receptor antagonists are currently being studied as adjuncts to opioids in order to reduce addictive and psychoactive effects while maintaining analgesia [54]. Ketamine has traditionally been used perioperatively for its analgesic and anesthetic properties. However, in recent years, ketamine infusions have been increasingly explored outside of the operating room for specific patients with complex regional pain syndrome and neuropathic pain [17]. Although more research is needed, initial findings suggest pain relief ranging from weeks to months [56,57]. Similarly, different institutions are exploring the role of lidocaine infusions that could provide short-term and long-term neuropathic pain relief [58].

Artificial intelligence is gaining momentum in medicine, with the potential to streamline complex administrative tasks and enhance patient analysis and treatment. Despite societal hesitance about AI in direct patient care, current data suggest that AI can aid in pain assessment, decision-making, and self-management [59]. Artificial intelligence can improve pain recognition and scoring through clinical note analysis and predict postoperative pain intensity using documentation and surveys, recommending optimal therapies. However, its effectiveness is limited by the variability in provider documentation. AI-based applications for pain self-management are promising as programs can track pain levels, frequency, and activity, offering tailored management strategies and feedback to providers [58]. For phantom limb pain management, virtual reality was found to significantly reduce pain in amputees when compared to augmented reality and mixed reality [60]. By leveraging AI applications, virtual platforms can enhance care coordination and patient satisfaction by facilitating connections among pain specialists, physical therapists, psychiatrists, and other providers. The PREVAIL program, an interdisciplinary pain management initiative delivered via telehealth at the Veterans Association, exemplifies a virtual program that could significantly benefit from integrating AI tools [61].

Whether providing care in-person or virtually, physicians must interact with patients in the state where they are licensed [62]. This limitation prevents providers from caring for out-of-state patients, further reducing the accessibility of care for rural Americans. With estimates as low as 8% of total pain specialists practicing in rural areas, there is a need to expand accessibility for rural Americans [9]. Policies that improve funding for both PCPs and residents in rural areas have the potential to both increase the utilization of care and diversify pain management techniques. Provider education in pain management may also reduce the amount of opioids prescribed and streamline the transition of care from acute to chronic pain. Project Extension for Community Healthcare Outcomes (ECHO) has shown success in closing the gap in pain education for providers pursuing continuing education [63]. Implemented in 2003 in New Mexico, the program has demonstrated significant value at both the community and patient levels, evidenced by a decrease in average hemoglobin A1c over five months [64]. Expanding funding for similar programs in rural areas could enhance patient care by equipping primary care providers and midlevel practitioners with the necessary training.

The U.S. medical system may also look to other countries for inspiration regarding successful chronic pain treatment options in rural populations. For example, the Chronic Pain Management Program (CPMP) at St. Joseph Care Group in Ontario, Canada, provides an example of how a multidisciplinary patient-centered model of chronic pain delivery can work to enhance the quality of delivered care [65]. All CPMP patients utilized a mixture of in-person and virtual appointments and met with physicians, social workers, psychologists, and other providers. This holistic approach enabled patients to receive individualized, multidisciplinary treatment that they might not have otherwise accessed, resulting in comprehensive and targeted treatment plans [65]. Another case report in Japan highlights a similarly utilized patient-centered multidisciplinary approach for treating an elderly female patient suffering from rheumatoid arthritis-related chronic pain [59]. The team considered barriers such as financial insecurity, living in a mountainous region, nondriving status, and limited public transportation, along with her goals of resuming daily activities, reducing treatment costs, and managing pain to create her treatment strategy. This program addressed the patient’s medical and social needs using an interdisciplinary team while respecting patient autonomy [59].

## 5. Conclusions

Chronic pain poses a pervasive and legitimate concern in the rural U.S. patient population. Not only are rural populations at greater risk of experiencing chronic pain, but they also experience increased challenges in accessing proper pain treatment. These barriers include a lack of pain medicine physicians and alternative pain providers, decreased utilization of available pain treatment modalities, inadequate provider knowledge of opioid prescription guidelines, and cultural differences such as increased distrust of the medical system. Rural populations are increasingly using nonmedical therapies and telehealth, although such utilization is still limited relative to urban populations. Strategies such as educating providers on proper opioid prescription, expanding telehealth where appropriate, continuing to value patient education and counseling, and improving access to different arms of pain medicine management show the most promise in improving the efficacy and accessibility of care. Research in pain management is still overall lacking in rural populations, and this paucity is further exacerbated within rural Black Indigenous People of Color (BIPOC) populations. Additional efforts should be undertaken to preserve access to telehealth, incentivize healthcare trainees to practice in rural areas, and support providers in following updated pain guidelines. 

## Data Availability

Data sharing is not applicable to this article as no new data were created or analyzed in this study.

## References

[1] Rikard S.M. (2023). Chronic Pain among Adults—United States, 2019–2021. MMWR. Morb. Mortal. Wkly. Rep..

[2] Zis P., Daskalaki A., Bountouni I., Sykioti P., Varrassi G., Paladini A. (2017). Depression and chronic pain in the elderly: Links and management challenges. Clin. Interv. Aging.

[3] Fishbain D.A., Lewis J.E., Gao J. (2014). The Pain Suicidality Association: A Narrative Review. Pain Med..

[4] Khalid S., Sambamoorthi U., Umer A., Lilly C.L., Gross D.K., Innes K.E. (2022). Increased Odds of Incident Alzheimer’s Disease and Related Dementias in Presence of Common Non-Cancer Chronic Pain Conditions in Appalachian Older Adults. J. Aging Health.

[5] Ditre J.W., Zale E.L., LaRowe L.R. (2019). A Reciprocal Model of Pain and Substance Use: Transdiagnostic Considerations, Clinical Implications, and Future Directions. Annu. Rev. Clin. Psychol..

[6] Rafferty A.P., Luo H., Egan K.L., Bell R.A., Gaskins Little N.R., Imai S. (2021). Rural, Suburban, and Urban Differences in Chronic Pain and Coping among Adults in North Carolina: 2018 Behavioral Risk Factor Surveillance System. Prev. Chronic Dis..

[7] Jensen L., Monnat S.M., Green J.J., Hunter L.M., Sliwinski M.J. (2020). Rural Population Health and Aging: Toward a Multilevel and Multidimensional Research Agenda for the 2020s. Am. J. Public Health.

[8] Cheeseman Day J., Hays D., Smith A. (2016). A Glance at the Age Structure and Labor Force Participation of Rural America. United States Census Bureau. https://www.census.gov/newsroom/blogs/random-samplings/2016/12/a_glance_at_the_age.html.

[9] Breuer B., Pappagallo M., Tai J.Y., Portenoy R.K. (2007). U.S. board-certified pain physician practices: Uniformity and census data of their locations. J. Pain.

[10] Smith T.J., Hillner B.E. (2019). The Cost of Pain. JAMA Netw. Open..

[11] Dahlhamer J., Lucas J., Zelaya C., Nahin R., Mackey S., DeBar L., Kerns R., Von Korff M., Porter L., Helmick C. (2018). Prevalence of Chronic Pain and High-Impact Chronic Pain among Adults—United States, 2016. Morb. Mortal. Wkly. Rep..

[12] United States Census Bureau In Some States, More Than Half of Older Residents Live in Rural Areas. Census.gov. https://www.census.gov/library/stories/2019/10/older-population-in-rural-america.html.

[13] Danilov A., Danilov A., Barulin A., Kurushina O., Latysheva N. (2020). Interdisciplinary approach to chronic pain management. Postgrad. Med..

[14] Ramsay-Seaner K., Letcher A., Hoffman M.S., Anderson E., Heckmann C. (2022). Perceptions of prescription opioid use among rural farming and ranching communities: Preliminary implications for outreach and treatment. Subst. Abuse.

[15] Nguyen L.H., Dawson J.E., Brooks M., Khan J.S., Telusca N. (2023). Disparities in Pain Management. Anesthesiol. Clin..

[16] Eaton L., Langford D., Meins A., Rue T., Tauben D., Doorenbos A. (2018). Use of Self-Management Interventions for Chronic Pain Management: A Comparison between Rural and Nonrural Residents. Pain. Manag. Nurs..

[17] Tauben D., Stacey B. (2024). Approach to the Management of Chronic Non-Cancer Pain in Adults. UpToDate. https://www.uptodate.com/contents/approach-to-the-management-of-chronic-non-cancer-pain-in-adults.

[18] Parchman M.L., Ike B., Osterhage K.P., Baldwin L.M., Stephens K.A., Sutton S. (2020). Barriers and facilitators to implementing changes in opioid prescribing in rural primary care clinics. J. Clin. Transl. Sci..

[19] Parlier A.B., Galvin S.L., Thach S., Kruidenier D., Fagan E.B. (2018). The Road to Rural Primary Care: A Narrative Review of Factors That Help Develop, Recruit, and Retain Rural Primary Care Physicians. Acad. Med..

[20] Decker S.A., Culp K.R., Cacchione P.Z. (2009). Evaluation of musculoskeletal pain management practices in rural nursing homes compared with evidence-based criteria. Pain Manag. Nurs..

[21] Elhakim M., Dexter F., Pearson A.C.S. (2019). US critical access hospitals’ listings of pain medicine physicians and other clinicians performing interventional pain procedures. J. Clin. Anesth..

[22] Rodgers-Melnick S.N., Trager R.J., Love T.E., Dusek J.A. (2024). Engagement in Integrative and Nonpharmacologic Pain Management Modalities among Adults with Chronic Pain: Analysis of the 2019 National Health Interview Survey. J. Pain Res..

[23] Day M.A., Thorn B.E. (2010). The relationship of demographic and psychosocial variables to pain-related outcomes in a rural chronic pain population. Pain.

[24] Gessert C., Waring S., Bailey-Davis L., Conway P., Roberts M., VanWormer J. (2015). Rural definition of health: A systematic literature review. BMC Public Health.

[25] Mares J.G., Lund B.C., Adamowicz J.L., Burgess D.J., Rothmiller S.J., Hadlandsmyth K. (2023). Differences in chronic pain care receipt among veterans from differing racialized groups and the impact of rural versus urban residence. J. Rural. Health.

[26] Qudah B., Maurer M.A., Mott D.A., Chui M.A. (2022). Discordance in Addressing Opioid Crisis in Rural Communities: Patient and Provider Perspectives. Pharmacy.

[27] Vallerand A.H., Fouladbakhsh J.M., Templin T. (2004). Self-Treatment of Pain in a Rural Area. J. Rural. Health.

[28] Prunuske J.P., Hill C.A.S., Hager K.D., Lemieux A.M., Swanoski M.T., Anderson G.W., Lutfiyya M.N. (2014). Opioid prescribing patterns for non-malignant chronic pain for rural versus non-rural US adults: A population-based study using 2010 NAMCS data. BMC Health Serv. Res..

[29] Kapoor S., Thorn B.E. (2014). Healthcare use and prescription of opioids in rural residents with pain. Rural Remote Health.

[30] Brunner W.M., Pullyblank K., Scribani M.B., Krupa N.L. (2022). Loneliness among Rural Self-Management Education Program Enrollees during the COVID-19 Pandemic. Am. J. Health Promot..

[31] Cheeseman Day J. (2019). Rates of Uninsured Fall in Rural Counties, Remain Higher than Urban Counties. United States Census Bureau. https://www.census.gov/library/stories/2019/04/health-insurance-rural-america.html.

[32] Aging in Rural Communities—PubMed. https://pubmed-ncbi-nlm-nih-gov.ezproxy.bu.edu/36404874/.

[33] Tul Y., Unruh A., Dick B.D. (2011). Yoga for chronic pain management: A qualitative exploration. Scand. J. Caring Sci..

[34] Bridges L., Sharma M. (2017). The Efficacy of Yoga as a Form of Treatment for Depression. J. Evid-Based Complement. Altern. Med..

[35] Manchikanti L., Malla Y., Wargo B., Pampati V., Fellows B. (2012). Complications of fluoroscopically directed facet joint nerve blocks: A prospective evaluation of 7500 episodes with 43,000 nerve blocks. Pain. Physician..

[36] Copenhaver D., Pritzlaff S., Jung M., Singh N. (2024). Interventional Therapies for Chronic Pain. UpToDate. https://www.uptodate.com/contents/interventional-therapies-for-chronic-pain?topicRef=126111&source=see_link#H62304578.

[37] A Chen J., DeFaccio R.J., Gelman H., Thomas E.R., Indresano J., Dawson T.C., Glynn L.H., Sandbrink F., Zeliadt S.B. (2022). Telehealth and Rural-Urban Differences in Receipt of Pain Care in the Veterans Health Administration. Pain. Med. Malden Mass..

[38] Spleen A., Lengerich E., Camacho F., Vanderpool R. (2014). Health Care Avoidance among Rural Populations: Results From a Nationally Representative Survey. J. Rural. Health.

[39] Mittinty M.M., Vanlint S., Stocks N., Mittinty M.N., Lorimer Moseley G. (2018). Exploring effect of pain education on chronic pain patients’ expectation of recovery and pain intensity. Scand. J. Pain.

[40] Products—Data Briefs—Number 372—August 2020. https://www.cdc.gov/nchs/products/databriefs/db372.htm.

[41] Ernst M., Niederer D., Werner A.M., Czaja S.J., Mikton C., Ong A.D., Rosen T., Brähler E., Beutel M.E. (2022). Loneliness before and during the COVID-19 pandemic: A systematic review with meta-analysis. Am. Psychol..

[42] Kong L.J., Lauche R., Klose P., Bu J.H., Yang X.C., Guo C.Q., Dobos G., Cheng Y.W. (2016). Tai Chi for Chronic Pain Conditions: A Systematic Review and Meta-analysis of Randomized Controlled Trials. Sci. Rep..

[43] Sani N.A., Yusoff S.S.M., Norhayati M.N., Zainudin A.M. (2023). Tai Chi Exercise for Mental and Physical Well-Being in Patients with Depressive Symptoms: A Systematic Review and Meta-Analysis. Int. J. Environ. Res. Public Health.

[44] Jain F.A., Walsh R.N., Eisendrath S.J., Christensen S., Cahn B.R. (2015). Critical Analysis of the Efficacy of Meditation Therapies for Acute and Subacute Phase Treatment of Depressive Disorders: A Systematic Review. Psychosomatics.

[45] Wu Y., Yan D., Yang J. (2023). Effectiveness of yoga for major depressive disorder: A systematic review and meta-analysis. Front. Psychiatry.

[46] la Cour P., Petersen M. (2015). Effects of Mindfulness Meditation on Chronic Pain: A Randomized Controlled Trial. Pain. Med..

[47] Felter C.E., Zalewski K., Jermann R., Palmer P.L., Baier A.E., Falvey J.R. (2022). Rural Health: The Dirt Road Less Traveled. Phys. Ther..

[48] Levy C.E., Silverman E., Jia H., Geiss M., Omura D. (2015). Effects of physical therapy delivery via home video telerehabilitation on functional and health-related quality of life outcomes. J. Rehabil. Res. Dev..

[49] Vorenkamp K.E., Kochat S., Breckner F., Dimon C. (2022). Challenges in Utilizing Telehealth for Chronic Pain. Curr. Pain Headache Rep..

[50] Tauben D.J., Langford D.J., Sturgeon J.A., Rundell S.D., Towle C., Bockman C., Nicholas M. (2020). Optimizing telehealth pain care after COVID-19. Pain.

[51] Strand N., Tieppo Francio V., Turkiewicz M., El Helou A. (2022). Advances in Pain Medicine: A Review of New Technologies. Curr. Pain Headache Rep..

[52] Oliveira M.F.D., Johnson D.S., Demchak T., Tomazoni S.S., Leal-Junior E.C. (2021). Low-intensity LASER and LED (photobiomodulation therapy) for pain control of the most common musculoskeletal conditions. Eur. J. Phys. Rehabil. Med..

[53] Ergonenc T., Stockman J. (2021). New ultrasound-guided techniques in chronic pain management: An update. Curr. Opin. Anesthesiol..

[54] LaVigne J.E., Alles S.R. (2022). CCK2 receptors in chronic pain. Neurobiol. Pain.

[55] Lamer T.J., Moeschler S.M., Gazelka H.M., Hooten W.M., Benden M.A., Murad M.H. (2019). Spinal Stimulation for the Treatment of Intractable Spine and Limb Pain: A Systematic Review of RCTs and Meta-Analysis. Mayo Clin. Proc..

[56] Xu J., Herndon C., Anderson S., Getson P., Foorsov V., Harbut R., Moskovitz P., Harden R.N. (2019). Intravenous Ketamine Infusion for Complex Regional Pain Syndrome: Survey, Consensus, and a Reference Protocol. Pain Med..

[57] Cohen S.P., Bhatia A., Buvanendran A., Schwenk E.S., Wasan A.D., Hurley R.W., Viscusi E.R., Narouze S., Davis F.N., Ritchie E.C. (2018). Consensus Guidelines on the Use of Intravenous Ketamine Infusions for Chronic Pain from the American Society of Regional Anesthesia and Pain Medicine, the American Academy of Pain Medicine, and the American Society of Anesthesiologists. Reg. Anesth. Pain Med..

[58] Iacob E., Hagn E., Sindt J., Brogan S., Tadler S.C., Kennington K.S., Hare B.D., E Bokat C., Donaldson G.W., Okifuji A. (2018). Tertiary Care Clinical Experience with Intravenous Lidocaine Infusions for the Treatment of Chronic Pain. Pain Med..

[59] Ohta R., Sano C. (2023). Integrating Clinical and Socio-Environmental Approaches in Managing Rheumatoid Arthritis with Social Determinants of Health: A Case Study of an Elderly Patient in Rural Japan. Cureus.

[60] Cheung J.C.-W., Cheung D.S.K., Ni M., Chen K.-W., Mao Y.-J., Feng L., Lam W.-K., Wong D.W.-C., Leung A.K.-L. (2023). X-reality for phantom limb management for amputees: A systematic review and meta-analysis. Eng. Regen..

[61] Darnall B.D., Edwards K.A., Courtney R.E., Ziadni M.S., Simons L.E., Harrison L.E. (2023). Innovative treatment formats, technologies, and clinician trainings that improve access to behavioral pain treatment for youth and adults. Front. Pain Res..

[62] Health Resources & Services Administration (2024). Licensing across State Lines. Telehealth.HHS.gov. https://telehealth.hhs.gov/licensure/licensing-across-state-lines.

[63] Katzman J.G., Comerci G., Boyle J.F., Duhigg D., Shelley B., Olivas C., Daitz B., Carroll C., Som D., Monette R. (2014). Innovative Telementoring for Pain Management: Project ECHO Pain. J. Contin. Educ. Health Prof..

[64] Gordon S.E., Monti S.M., Catic A.G., Dufour A.B., Mattison M., Morgan M., Lipsitz L.A. (2015). Project ECHO-AGE and Nursing Home Quality of Care. J. Am. Med. Dir. Assoc..

[65] Shojaei H., Lakha S.F., Lyon A., Halabecki M., Donaghy M., Mailis A. (2022). Evolution of a chronic pain management program in a Northwestern Ontario community: From structural elements to practical application. BMC Health Serv. Res..

